# Involving patients and clinicians in the development of a randomised clinical trial protocol to assess spinal manual therapy versus nerve root injection for patients with lumbar radiculopathy: a patient and public involvement project to inform the SALuBRITY trial design

**DOI:** 10.1186/s40900-023-00536-0

**Published:** 2024-01-17

**Authors:** Corina Ryf, Léonie Hofstetter, Lauren Clack, Cesar A. Hincapié

**Affiliations:** 1 Department of Chiropractic Medicine, Balgrist University Hospital, University of Zurich, Zurich, Switzerland; 2 EBPI-UWZH Musculoskeletal Epidemiology Research Group, Balgrist University Hospital, University of Zurich, Zurich, Switzerland; 3 Head of Musculoskeletal Epidemiology Research, Epidemiology, Biostatistics and Prevention Institute (EBPI) & University Spine Centre Zurich, Balgrist University Hospital, University of Zurich, Zurich, Switzerland; 4 University Spine Centre Zurich (UWZH), Balgrist University Hospital, University of Zurich, Zurich, Switzerland; 5 Institute for Implementation Science in Health Care, University of Zurich, Zurich, Switzerland; 6 Department of Infectious Diseases and Hospital Epidemiology, University Hospital Zurich, Zurich, Switzerland

**Keywords:** Patient and public involvement, Patient engagement, Research design, Lumbar radiculopathy, Sciatica, Back pain, Spinal manual therapy, Chiropractic, Corticosteroid nerve root injection, Outcome assessment

## Abstract

**Background:**

Spinal manual therapy and corticosteroid nerve root injection are commonly used to treat patients with lumbar radiculopathy. The SALuBRITY trial—a two parallel group, double sham controlled, randomised clinical trial—is being developed to compare their effectiveness. By gathering patients’ and clinicians’ perspectives and involving them in discussions related to the trial research question and objectives, proposed trial recruitment processes, methods, and outcome measures, we aimed to improve the relevance and quality of the SALuBRITY trial.

**Methods:**

We involved patients with lived experience of lumbar radiculopathy (n = 5) and primary care clinicians (n = 4) with experience in the treatment of these patients. Involvement activities included an initial kick-off event to introduce the project, establishing a shared purpose statement, and empowering patient and clinician advisors for their involvement, followed by semi-structured group and individual interviews, and questionnaires to evaluate the experience throughout the project.

**Results:**

Both patient and clinician advisors endorsed the significance and relevance of the trial’s objectives. Patients assessed the proposed trial methods as acceptable within the context of a trusting patient-clinician relationship. A trial recruitment and enrolment target time of up to five days was regarded as acceptable, although patients with chronic radiculopathy may need more time to consider their trial participation decision. All advisors reached consensus on the acceptability of a medication washout phase of 12- to 24-h before pain outcome measurement, with the inclusion of a rescue medication protocol. Both advisory groups preferred leg pain over back pain as the primary clinical outcome, with patient advisors advocating for personalized primary pain localization. Furthermore, patients requested expanding the pain, enjoyment, and general activity scale with peak pain intensity, rather than average pain alone. Patient and clinician advisors evaluated their engagement in clinical research as meaningful and impactful.

**Conclusion:**

Patient and public involvement resulted in important and relevant considerations for the SALuBRITY trial, spanning all research phases. These findings hold promise for enhancing the trial’s quality and relevance and improving its translation into clinical practice.

**Supplementary Information:**

The online version contains supplementary material available at 10.1186/s40900-023-00536-0.

## Abstract (highlights)

In the SALuBRITY trial, we are comparing the effectiveness of two common treatments, spinal manual therapy and corticosteroid nerve root injection, for patients with lumbar radiculopathy, which is back pain that spreads into the leg because of an irritated nerve in the lower back. To ensure the trial's relevance and quality, we actively engaged both patients with lived experience of lumbar radiculopathy and primary care clinicians. Their valuable input supported the trial's objectives and found the proposed methods acceptable, especially in the context of a trusting patient-clinician relationship. Key consensus points included a reasonable recruitment and enrollment processing target time of up to five days, though chronic radiculopathy patients may need more time. A 12- to 24-h medication washout phase before pain assessment was endorsed, with emphasis on a rescue medication option. Leg pain was preferred over back pain as the primary outcome, and patients advocated for personalizing primary pain location. Our advisors evaluated their engagement in this patient and public involvement project as meaningful and impactful. Involving patients and clinicians in the SALuBRITY trial development process promises to enhance the trial's quality, relevance, and translation into clinical practice, ensuring it addresses the real needs and experiences of patients with lumbar radiculopathy.

**Supplementary Information:**

The online version contains supplementary material available at 10.1186/s40900-023-00536-0.

## Background

Patient and public involvement (PPI) refers to the involvement of people with lived experience of a health condition, as well as the wider public, in different stages of the research process, ensuring that research is relevant, acceptable, and ultimately more likely to lead to improvements in health outcomes [1]. Around the world, there are patient groups [2] and centres of excellence [3, 4], that are interested in joint research action, establishing networks [5], and providing guidance for partnering with patients, health and care professionals, and industry partners [3, 6–8]. More recently, many research funding calls are requiring meaningful PPI work as part of the development or process of project proposals for funding. [6, 9]

PPI can help to identify possible challenges in the collaboration of researchers with patients and all study partners throughout the research process, with potential benefits for patients and all involved stakeholders. Patients describe advantages such as empowerment, increased knowledge, and confidence, which emphasize the wide societal benefits and the potential for research to act as a positive force in society. Researchers may benefit from project development that is more relevant to end-users, the conceptualization of research project proposals that are more compelling and likely to secure funding [10, 11], better participant enrolment rates [10, 12], and increased trust and advocacy within the study population community [13].

In this report, we describe a PPI project that aimed to enhance the quality and relevance of a future randomised clinical trial to assess spinal manual therapy versus corticosteroid nerve root injection for the management of patients with lumbar radiculopathy. Lumbar radiculopathy is a condition characterized by low back pain that radiates down the leg in a lumbar spine nerve distribution and is clinically indicative of irritation or compression of a lumbar spine nerve root [14]. Conservative therapeutic approaches such as spinal manual therapy [15–17] and corticosteroid nerve root injections [18] are frequently used for the treatment of patients with lumbar radiculopathy. Despite their common use, there remains uncertainty regarding the comparative effectiveness of spinal manual therapy and nerve root injections. To address this knowledge gap, the SALuBRITY trial is being developed—a two parallel group, double sham controlled, randomized clinical trial.

By involving patients with lived experience of lumbar radiculopathy and primary care clinicians that care for such patients in the development of the SALuBRITY trial, we aimed in this PPI project to answer the following questions:

- 1: Is the SALuBRITY trial’s main question and objective important and relevant to patients with lumbar radiculopathy and primary care clinicians of patients with lumbar radiculopathy?
- 2: Are the recruitment process and proposed methods for the clinical trial acceptable and sensitive to potential participants and clinician collaborators?
- 3: Are the proposed trial outcomes important and relevant to patients with lumbar radiculopathy?
- 4: Are the language and content of trial information appropriate and accessible to participants and clinicians?
- 5: What is the impact of PPI on the relevance and quality of the SALuBRITY randomized clinical trial?

## Methods

### Design

The protocol for this PPI project was published previously [19]. This qualitative study was informed by the Critical Outcomes of Research Engagement (CORE) framework [20], with the intention of partnering with advisors across different stages of research. The CORE framework was adapted for the SALuBRITY trial (see Additional file 1: Figure S1), providing a structure for organizing PPI inputs throughout the research stages proposing desired outcomes and appropriate methods. The Guidance for Reporting Involvement of Patients and the Public (GRIPP2) long form checklist [21] was used as a framework for reporting this PPI project (see Additional file 2: Table S1). Patients or members of the public involved in planning or advising on research are not acting in the same way as research participants. They are acting as specialist advisers, providing valuable knowledge and expertise based on their experience of a health condition or public health concern. The independent research ethics committee of Canton Zurich confirmed that ethical approval was not required for this PPI project.

### Patient and clinician advisors

Purposeful sampling was used to recruit a small group of patient and clinician advisors, where clinicians represent the “public” in PPI, based on prespecified eligibility criteria while attempting to involve advisors of a variety of ages, duration of symptoms (patients), years of clinical work experience (clinicians), and a mix of both men and women. Besides a small token of appreciation (30 CHF gift card) at project completion, there was no monetary or other form of compensation of all PPI advisors.

We invited patient advisors aged between 18 and 65 years, with lived experience of lumbar radiculopathy and treatment with spinal manual therapy or corticosteroid nerve root injection, and consenting to be involved as a patient advisor. We preferred patients who had experience with multiple treatments for radiculopathy (such as chiropractic treatment, physiotherapy, massage, nerve root injection, or surgery).

Primary care clinicians in the surrounding region of Zurich were contacted and informed about the PPI project. They were considered eligible if they had clinical work experience providing healthcare to patients with lumbar radiculopathy and were willing to be involved.

### PPI activities

All PPI activities were carried out virtually via Zoom due to the COVID-19 pandemic. After distribution of a demographic questionnaire, an initial kick-off event was conducted to prepare and empower the advisors for the involvement in the PPI project [19]. During this event, information on the SALuBRITY trial was provided to all advisors, followed by familiarization with planned PPI project tasks in two separate groups. The expectations of all project partners were collected, combined to formulate a shared purpose, and fed back to all participants to ensure an accurate interpretation. Afterwards, a patient focus group and individual semi-structured clinician interviews were conducted. Brief vignettes were developed [19] covering key PPI topics, subsequently integrated into distinct interview guides tailored for patient and clinician advisors. Open questions were used to initiate discussions, complemented by pre-defined structured questions for potential discussion recalibration.

Additional patient advisors fulfilled a think-aloud task to collect feedback in participant information document until data saturation was reached. Data saturation involves recognizing informational redundancy in the data, and it can be detected early on in the process, distinct from and preceding formal data analysis [22]. Task instructions adhered to a predetermined protocol, which was communicated to the participants before commencement. They were repeatedly directed to articulate their thoughts aloud without the concern of interruption.The instruction guide was published in our protocol [19]. To evaluate advisors’ and researchers’ experience of the PPI project, two questionnaires were modified for use in this PPI project and provided to all PPI participants.

### Data collection and analysis

An electronic demographic questionnaire was distributed after recruitment. Individual clinician interviews were moderated in English (CAH), while the patient focus group interview was moderated in German (LH) using the vignettes to guide the interviews. Two members of the research team took comprehensive notes during the advisor interviews (LC and CR). The notes were consolidated in an interview summary (i.e., member checking document), which was then shared with interview participants in a member checking process [23]. Any discrepancies were resolved by discussion with the patient and clinician advisors to ensure accurate interpretation of their input and perspectives [23]. The patient focus group member checking document was translated from German into English. All interviews were digitally recorded to enable subsequent access if needed.

The qualitative data from the member checking documents were evaluated using thematic analysis [24]. They were manually coded using the vignettes as guidance for deductive thinking. If the member checking content did not align with any of the predefined vignettes, an inductive approach was adopted to elicit a more comprehensive perspective on the topics of interest. Two members of the research team (LC, CR) performed this independently. The codes of the patient group and the individual clinician interviews were merged to a basic coding notebook, a working document composed of categories with a brief description, representing the foundation of the systematic coding. For this process, two members of the research team (LH, CR) applied the basic codes throughout the member checking documents, finalizing the coding notebook by generating subcategories, categories, and overarching themes (see Additional file 3: Table S2).

Building on the CORE framework [20], every research stage was extended by desired outcomes revealing it’s overarching impact on each stage, both reflecting an interplay between advisors’ statements and our own further interpretation as researchers. Subsequent analysis resulted in the formulation of PPI generated considerations for the SALuBRITY trial.

## Results

### Characteristics of patient and clinician advisors

Initially, four patients and four clinicians were recruited for this PPI project. One patient could not attend the kick-off meeting and was excluded from further PPI activities. For the think-aloud task, another two patients were recruited after which feedback saturation was achieved, resulting in five patient and four clinician advisors (n = 9) in total. Table 1 provides a summary of patient and clinician advisor characteristics. All patients presented with chronic symptom duration (> 3 months) and all had prior experience with chiropractic treatment and physiotherapy. Additionally, four patients had received pain medication and corticosteroid injections, while one patient had undergone previous back surgery. Three of the clinicians were general practitioners and one was an orthopaedist working as a musculoskeletal specialized primary care physician in a multidisciplinary medical centre. All had 10 or more years of practical experience and endorsed seeing less than four patients with lumbar radiculopathy per month, except the orthopaedist who reported seeing 4–8 patients with lumbar radiculopathy per month.

**Table 1:** Characteristics of patient and clinician advisors

Patient advisor	Sex	Age (y)	Profession	Symptom duration	Treatment experience
P1	Male	65	Retiree	> 2 years	C, I, M, P
P2	Male	30	Scientific assistant	> 2 years	C, I, M, P, S
P3	Female	57	Architect	6–12 months	C, P
P4	Male	27	Software engineer	> 2 years	C, I, M, P
P5	Male	42	Art blacksmith	> 2 years	C, I, M, P

Clinician advisor	Sex	Age	Profession	Clinical work experience	N patients seen with lumbar radiculopathy
C1	Female	38	GP	10 to 20 years	< 4 per month
C2	Male	66	GP	> 20 years	< 4 per month
C3	Female	68	GP	> 20 years	< 4 per month
C4	Male	36	Orthopaedist	10 to 20 years	4 to 8 per month

*C* chiropractic, *I* corticosteroid infiltration, *M* pain medication, *N* number, *P* physiotherapy, *S* spine surgery, *GP* general practitioner, *y* years

### Shared purpose

All advisors agreed on the following four main concepts encompassed within the shared purpose statement: (1) the exchange of personal life experiences, (2) sharing of research insights, (3) focusing on patient-centred perspectives, and (4) being open to exploring different treatment modalities. An overarching goal was established to compare the indications and effectiveness of treatment methods, along with broad dissemination of new knowledge (see Additional file 4: Figure S2).

### PPI generated considerations for the SALuBRITY trial

**Fig. 1:**
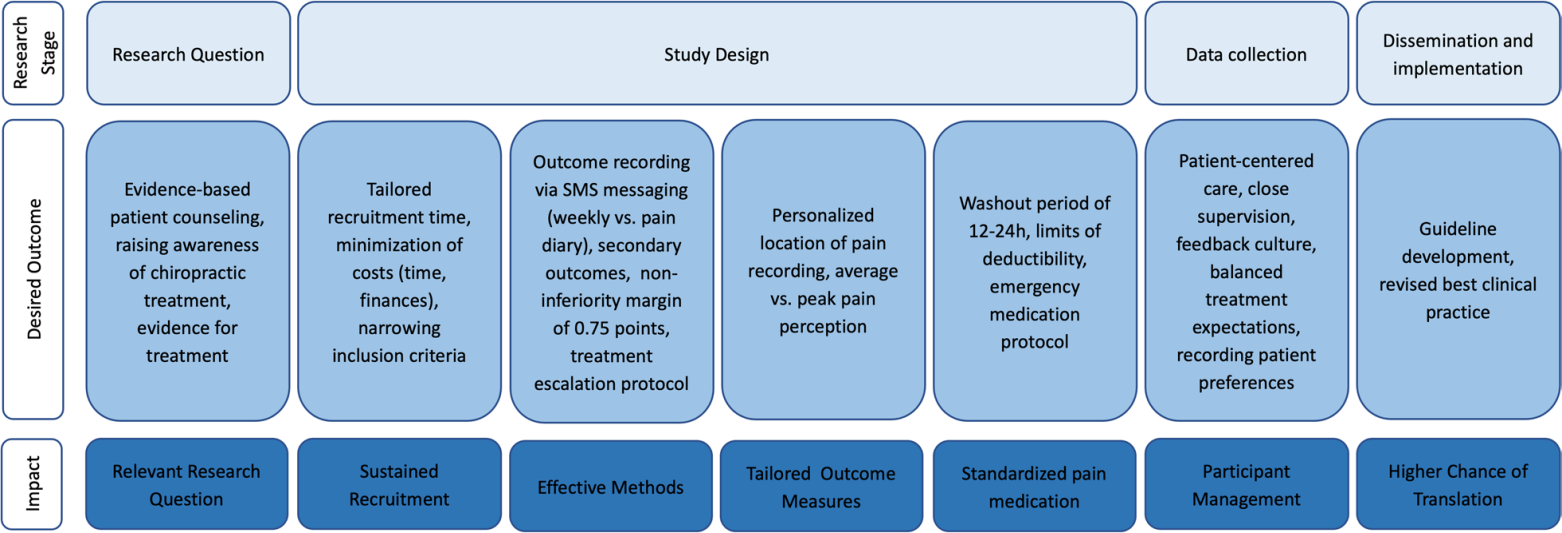
Research stage, desired outcomes, and potential impacts of PPI activities on future SALuBRITY trial, adapted from the CORE framework

Figure 1 outlines potential impacts and desired outcomes related to the design and development of the SALuBRITY trial raised through PPI activities and organized according to CORE framework [20] research stages.

#### Research question

All advisors agreed on the importance and relevance of the SALuBRITY trial’s main question and objective and expressed optimism about the potential positive outcomes (e.g., short term treatment option, personalized treatment plan, increased evidence and understanding of both chiropractic and nerve root injection treatments) that could arise from conducting the trial. The study results should support clinicians in making informed decisions regarding the appropriate therapeutic option for patients, thereby facilitating high-quality sustainable patient therapy counselling.

“The outcomes of this study are intended to assist a general practitioner in identifying the appropriate treatment option for each individual patient.” P1

Furthermore, patients expressed their desire to increase awareness of non-pharmacological spinal manual therapy as a potential early, non-invasive, conservative treatment option.

“A substantial number of patients experiencing back pain are primarily referred to physical therapy, while lacking awareness of the potential benefits of chiropractic care.” P3

#### Study design: participant information document

The participant information documents were presented in a suitable and comprehensible manner for the patient advisors (research question 4). Minor feedback was provided concerning the utilization of technical terminology commonly found in clinical studies (e.g., "sponsor"), as well as the consistency of certain terms (such as "infiltration" vs. "injection"). In terms of content, one patient found it difficult to justify paracetamol as the trial rescue medication due to personal experience of paracetamol being ineffective in alleviating their radicular pain.

#### Study design: recruitment

Patient advisors emphasized the importance and value of offering potentially equivalent treatments in terms of effectiveness, benefits, and harms across both trial intervention groups, while also advocating against the use of untested experimental interventions. In terms of the proposed target time (up to 5 days) for patient recruitment to the trial, eligibility screening, consenting, enrolment, and trial treatment initiation, patient advisors highlighted the importance of considering patients’ needs and their pain severity. It was pointed out that patients suffering from a chronic radiculopathy may require more time to consider and decide on whether they would like to participate in the trial.

Clinician advisors suggested narrowing the trial inclusion criteria by excluding patients who experienced extreme levels of pain intensity. From clinicians’ perspective, the economic implication of loss of patients referred to the trial was identified as a possible barrier for recruiting clinicians, considering the potential financial loss for subsequent healthcare visits transferred to the trial clinicians rather than the recruiting clinician.

“What is the benefit for GPs? The financial aspects become relevant when the patient is referred to Balgrist [university hospital] and subsequent treatments are covered…” C4

#### Study design: methods

Clinician advisors found the proposed minimal important difference of 1 point on the pain intensity numerical rating scale between 0 and 10, and the proposed non-inferiority margin of 0.75 points acceptable. Furthermore, patient advisors supported the implementation of a double-sham-controlled protocol (i.e., a trial design feature where each intervention group has both an active/real treatment plus a sham/placebo treatment) in the presence of a trusting and positive patient-clinician relationship. Despite advisors’ overall comfort with the idea of using SMS messaging to collect trial outcomes, there were varying opinions regarding the preferred frequency of such SMS data collection messages over the study duration. Weekly SMS assessments were considered feasible, and daily pain diary monitoring was raised as a possibility that might be deemed acceptable by trial participants. Additionally, patient advisors endorsed the need for a treatment escalation or cross-over protocol in case participants do not respond sufficiently to the trial interventions and require stepped-up healthcare service:

“Is there an escalation protocol included? If the health condition does not improve or worsens, the participant should have the possibility to change treatment group.” P2

#### Study design: outcome measures

Differences among the advisors were most prominent when focusing on the trial outcomes. First, we learned that patient advisors evaluated treatment success not only based on their average pain levels, but also on the intensity of their peak pain. For one patient, back pain represented the most relevant pain localization together with numbness and paraesthesia of the leg. Three clinicians and two other patients endorsed leg pain intensity as the most relevant primary clinical outcome. Nonetheless, they all acknowledged the importance of evaluating overall pain (leg and back pain) to adequately characterize the entire patient population. One clinician advocated for separately assessing leg and back pain.

“Leg pain was more relevant; I also visited the doctor because of pain in the leg.” P2

In sum, the advisors suggested two essential outcomes, wherein leg pain was favoured over back pain, and overall pain was considered necessary to encompass all. Patients reached a consensus concerning the significance of secondary outcomes, as both the direct experience of pain and its impact on daily activities and quality of life were deemed noteworthy and important. In addition, they supported the potential secondary outcomes of reinstating mobility and alleviating psychological distress.

#### Study design: discontinuation of pain medication

The notion of asking that trial participants discontinue their pain medication prior to pain assessments challenged patient advisors. Although patients were supportive of a medication washout period of 12–24 h, they identified individual pain tolerance, restrictions in daily life, and contextual factors such as family and work responsibilities as factors that may influence the acceptability of this proposal. One clinician suggested that discontinuing medication for up to 48 h with alternative methods of pain management and good care would be reasonable, while another clinician advocated for a pragmatic approach of not discontinuing medication but documenting medication intake in detail. Furthermore, patient advisors expressed the need for an emergency medication protocol to manage intolerable pain during the discontinuation of medication.

#### Data collection

Characteristics suggested as important for achieving patient-centred clinical research and data collection were: establishing good patient-clinician rapport and connection, closely supervising and supporting during discontinuation of pain medication, and allowing participants to continue to see their doctor (the recruiting clinician) during trial participation if needed. Patient advisor interviews revealed that they often held preconceived notions regarding their medical condition and had preferences regarding treatment options.

“Patient profile for study? Purely emotional, I would decline participation if I had 50% chance of receiving a treatment I don't want (infiltration).” P3

Clinician advisors endorsed the value of receiving initial feedback/communication regarding study inclusion of their referred patients, as well as regular updates on patient and study progress throughout the duration of the study.

#### PPI considerations for SALuBRITY trial

Table 2 summarises the considerations for the SALuBRITY trial resulting from this PPI project, targeting several stages of the research process. First, the formulation of the participant information document requires careful consideration not only of study design and treatment details but also of patient expectations. Addressing patient and clinician costs becomes imperative in the recruitment phase. Furthermore, attention is directed towards trial methodologies and recommendations for optimizing clinician interactions.

**Table 2:** PPI considerations for the SALuBRITY trial

Target	Category	Sub-category	Considerations for SALuBRITY trial
Participant information document	Design	Patient-clinician relationship	Enable single point of contact for patients throughout trial participation
		Patient-centred care	Prioritize patient well-being
		Escalation protocol	Develop treatment cross-over protocol Establish study emergency withdrawal protocol
		Randomization	Record patient treatment preference
		Blinding	Ensure minimal exposure to unnecessary treatment
	Treatment	Expectations	Assure equal treatment methods across both trial intervention arms Ensure no use of untested experimental interventions
		Information	Inform about possible varying response to treatment Describe efforts to prevent negative treatment effects
		Care	Establish sufficient support during trial participation Offer experienced chiropractor treatment delivery
Recruitment	Clinician costs	Time	Avoid time-consuming recruiting process Simplify recruitment documents
		Finances	Allow ongoing patient care by recruiting clinician during trial participation
	Patient costs		Offer tailored trial recruitment and enrolment time (e.g., acute vs. chronic radiculopathy) Minimize the number of in-person study visits
	Recruitment process		Restrict participation to relevant range of pain intensity (e.g., 4–7 out of 10 on pain NRS) Align treatment initiation urgency with pain intensity
Outcome	Documentation		Measure and document additional medications, therapies, and activities
	Outcome measures		Record average and peak pain intensity Include mobility, quality of life, and psychological stress as secondary outcomes Individualize primary outcome pain location and document on trial enrolment Improve PEG scale comprehension by explaining “average”, providing examples for “pain interference on general activities”, and incorporating a visual aid
Pain medication	Discontinuation		Adopt a pragmatic approach for pain medication discontinuation Offer alternative pain treatment Develop emergency medication protocol
Clinician communication, engagement, and interaction			Contact potential clinician collaborators in person Provide feedback/status updates on trial events and milestones concerning their referred patient Implement study reminders (e.g., monthly via mail/newsletter) and disseminate findings/results

*NRS* numeric rating scale, *PEG scale* pain, enjoyment of life and general activity scale

#### Dissemination and implementation

Both clinicians and patients alike supported the concepts of comprehensive, impartial, and evidence-informed patient counselling and decision-making. The lack of understanding concerning the differences in benefits and harms between spinal manual therapy and corticosteroid nerve root injection was acknowledged, despite the common use of these treatments for the management of persons experiencing lumbar radiculopathy. One clinician reported applying manual techniques on patients with low back pain and finding nerve root injections to be effective as an escalating treatment for patients with lumbar radiculopathy who do not respond to manual therapies.

### Evaluation of advisors’ PPI experience

Three patients and all four clinicians (n = 7) were included in the evaluation of this PPI project. One survey was delivered just after the kick-off meeting, the other after completion of all PPI activities. The two patients performing the think-aloud method on the participant information document did not participate in the kick-off meeting and were not part of the evaluation. Most of the advisors (n = 6) felt very comfortable speaking up during the meetings and in their understanding of the project. However, two of the three patients did not feel as equipped to contribute to the PPI project as the other advisors (see Additional file 2: Table S1). One participant expressed a desire for more information prior to their involvement, specifically requesting a “summary of current research” when asked for suggestions on how to improve the project. They all had the impression of having a meaningful impact on the design and development of the future SALuBRITY trial. Clinician advisors emphasized the values of “providing my perspective”, “collaborating with GPs”, and “clinical experience” to be their most meaningful contributions. “Finding time” was perceived as the major challenge when partnering with researchers. As part of a multidisciplinary team, a few lessons learned by clinicians included the importance of “preparing to acquire a common language” and “avoiding preconceived ideas”. Patients found the most interesting lessons to be related to “differences in perspectives” and the “challenge of quantifying pain”.

## Discussion

### Summary of main findings

Both patient and clinician advisors acknowledged the importance and relevance of the objectives of the future SALuBRITY trial (research question 1) and advocated for evidence-based patient counselling and decision-making regarding treatment options for lumbar radiculopathy. Randomization, patient, and clinician blinding were deemed acceptable by advisors within the context of a trusting and positive patient-clinician relationship. A target time for trial recruitment and enrolment processing of up to five days was perceived as acceptable, although extension might be necessary for a chronic radiculopathy patient population, allowing for careful deliberation in their trial participation decisions (research question 2). All advisors reached consensus on implementing a medication washout phase lasting 12–24 h before outcome assessment, with the inclusion of an emergency medication protocol (e.g., graded scheme of pain analgesics ranging from weak to strong medication). Leg pain was favoured over back pain as the primary clinical outcome among both advisor groups, with patients advocating for the inclusion of average as well as peak pain intensity perception using the PEG scale (research question 3). All patients felt comfortable reporting clinical outcomes via SMS, but they did not reach consensus regarding its frequency (e.g., daily vs. weekly). Patient and clinician advisors evaluated their engagement in clinical research and this PPI project as meaningful and impactful (research question 5).

### Factors affecting patient participation

Patient recruitment and retention has been shown to be one of the most challenging aspects in the execution of clinical trials [12]. We have identified criteria potentially interfering with patient decisions (i.e., barriers) to be a study participant. Since the participant information document serves as the initial point of contact for potential participants with the SALuBRITY study, Table 2 highlights crucial elements to be considered for a comprehensible and patient-friendly document. Patient advisors supported potentially equivalent treatments across both trial intervention arms with respect to benefits and harms and sought assurance that untested experimental treatments should be avoided. To balance treatment expectations, these aspects should be adequately described in the participant information document. Additionally, they endorsed developing a cross-over treatment protocol in case of inadequate response or worsening of symptoms with the allocated trial treatment. Patient preferences are another factor influencing a patient’s decision to participate in a clinical trial. This is in line with previous work by Houghton and colleagues [25], arguing that treatment preferences may be key factor in a patient’s decision-making process. We learned through discussion with patients, that preferences may exist not only for trial treatments, but also for emergency medications, recruitment time, and location of perceived pain measurement. Assessment of all patient preferences warrants consideration at the time of study entry.

Monetary compensation was interpreted to be a thoughtful acknowledgment rather than an overly influencing factor for participation, although the belief in “own benefit” is part of the conceptual model developed by Houghton et al. [25] In the context of our PPI project, it was predominantly clinician advisors that raised concerns about potential cost implications. However, aligning with the conceptual model [25], it would be important to adequately account for expenses incurred by patients during trial participation and minimize non-essential time-related costs for all connected to the trial.

### Trial recruiting clinician network: collaboration and economic implications

To recruit enough patients for the trial, it is essential to establish a network of clinician collaborators involved in identifying and recruiting potentially eligible patients to the trial. Clinician advisors emphasised the importance of cost implications due to the loss of continued patient care by the recruiting clinician (Table 2). A clinician recruitment process that is efficient, low burden, and at least revenue neutral (i.e., no financial loss) was endorsed as highly relevant. This aligns with prior work, identifying barriers to providers’ referral of patients, including financial and time related burden [26] along with a knowledge gap (e.g., concepts and methods of clinical trials) [27, 28], problems with evaluating study protocols [27], concerns about patient health management and questions regarding the direct benefits of research participation for patients and recruiting clinicians [26]. Strategies to alleviate these barriers include simplifying standardized recruitment protocols [28], sufficient good quality training [27], and cultivating a broader comprehension of health research in general [28]. Our PPI project also revealed the importance of routine study reminders, feedback and communication with recruiting clinician collaborators concerning their patient’s status in the trial, research progress, and the dissemination of study findings.

### Implications for future research and PPI work

A primary objective of this project was to ascertain whether the proposed trial outcomes were considered relevant and meaningful by patients with lumbar radiculopathy and their treating clinicians. While both patient and clinician advisors endorsed a preference for leg pain over back pain as the primary outcome, patients advocated for personalizing pain assessment by allowing participants to choose their most relevant pain location (e.g., leg pain more disabling or back pain more disabling, Table 2). However, the implementation of personalized primary outcome is scientifically infeasible due to the negative implications on statistical power and primary outcome interpretation and comparability. This generates important questions about the implementability of findings derived from PPI work, revealing the possibility of discrepancies between PPI considerations and the scientific realities of trial design and execution. While researchers express concerns about upholding methodological rigor [29], patients emphasize the necessity for earlier involvement in the research lifecycle to propose constructive modifications and help address their lack of familiarity with research methods [12]. Prior literature also advocated for PPI initiated during earlier phases of trial planning, asserting an augmented influence through the comprehensive inclusion of patient’s perspective transcending mere information provision [12]. Engaging patients and the public in the early development of trial proposals, often corresponding to the grant-seeking phase of a research project, can also be challenging due to budget and fair compensation implications. The risk of a vicious cycle of PPI best practices and demands, coupled with financial and time constraints, can be high—this warrants consideration and attention by funding associations and research policymakers.

### Strengths and limitations

To our knowledge, this is one of the first PPI projects embedded in a randomized clinical trial investigating the effectiveness of conservative treatment options for patients with lumbar radiculopathy. Numerous frameworks exist for guiding patient involvement in health research, but their transferability is often limited. [30] Following the recommendation of Greenhalgh and her colleagues [30], we adapted the CORE framework [20] to enhance its applicability, thereby increasing the transferability of frameworks in PPI research.

Our PPI project has some limitations. First, it is possible that we may have missed collecting important advisor perspectives that were not queried. Although we employed tools to evaluate the impact of PPI and track the experience of our advisors, a broader evaluation [31] may have better captured potential negative impacts and long-term outcomes. While general practitioners and orthopedic specialists are often the first point of contact for many patients with back pain, our selection of clinician advisors does not encompass all musculoskeletal specialists. Second, certain inputs from advisors (e.g., a personalized primary outcome) cannot be considered for the SALuBRITY trial owing to methodological and resource limitations. Third, we acknowledge the use of a small sample size of PPI advisors (n = 9), although this is not unusual in qualitative research and we were guided by the concept of data saturation [32]. Fourth, we did not use full verbatim transcripts in our PPI data analysis. However, we observed no apparent disadvantages in relying on detailed notetaking and member-checking approaches and benefitted from full video and audio recordings of the PPI activities conducted online through Zoom. Fifth, data collection was conducted online during the COVID-19 pandemic, potentially limiting the experience of some advisors as part of the research team, while others may have appreciated the time- and commute-saving approach.

## Conclusion

This PPI project identified relevant considerations for a future clinical research trial across the spectrum of research stages and highlighted aspects from patient and clinician end-user perspectives that may improve the design and development of the SALuBRITY trial protocol. Patient and clinician advisors valued their contributions and involvement as meaningful and worthwhile. This work has the potential to enhance the relevance and quality of the SALuBRITY trial, ultimately improving its translation into clinical practice and the management of patients with lumbar radiculopathy.

### Supplementary Information

**Additional file 1. Figure S1: **Stages, outcomes, and methods of involvement.

**Additional file 2. Table S1: **GRIPP2 long form.

**Additional file 3. Table S2: **Themes, categories, and definitions with summarized coding examples.

**Additional file 4. Figure S2**: Shared purpose statement.

## Data Availability

The original records and the member checking documents are available on reasonable request.

## Abbreviations

C: Chiropractic
CORE: Critical outcomes of research engagement
GP: General practitioner
GRIPP2: Guidance for reporting involvement of patients and the public
I: Corticosteroid infiltration (nerve root injection)
M: Pain medication
NRS: Numeric rating scale
P: Physiotherapy
PEG scale: Pain, enjoyment of life and general activity scale
PPI: Patient and public involvement
S: Spine surgery
