# Supplementary material for: Involving patients and clinicians in the development of a randomised clinical trial protocol to assess spinal manual therapy versus nerve root injection for patients with lumbar radiculopathy: a patient and public involvement project to inform the SALuBRITY trial design
Source: Res Involv Engagem. 2024 Jan 17;10:8. doi: 10.1186/s40900-023-00536-0 (PMC10792780; doi:10.1186/s40900-023-00536-0)
Supplement: Supplementary file 1 — Additional file 1. Figure S1: Stages, outcomes, and methods of involvement. [file 40900_2023_536_MOESM1_ESM.docx]

## Additional file 1: eFigure 1. Stages, outcomes, and methods of involvement

Ryf C, Hofstetter L, Clack L, Hincapié CA. Involving patients and clinicians in the development of a randomised clinical trial protocol to assess spinal manual therapy versus nerve root injection for patients with lumbar radiculopathy: a patient and public involvement project to inform the SALuBRITY trial design (2023).

## eFigure 1. Stages, outcomes, and methods of involvement

Based on adaptations from the Critical Outcomes of Research Engagement (CORE) framework by Dillon et al. (2017) for measuring the impact of patient and lay involvement in research.

**
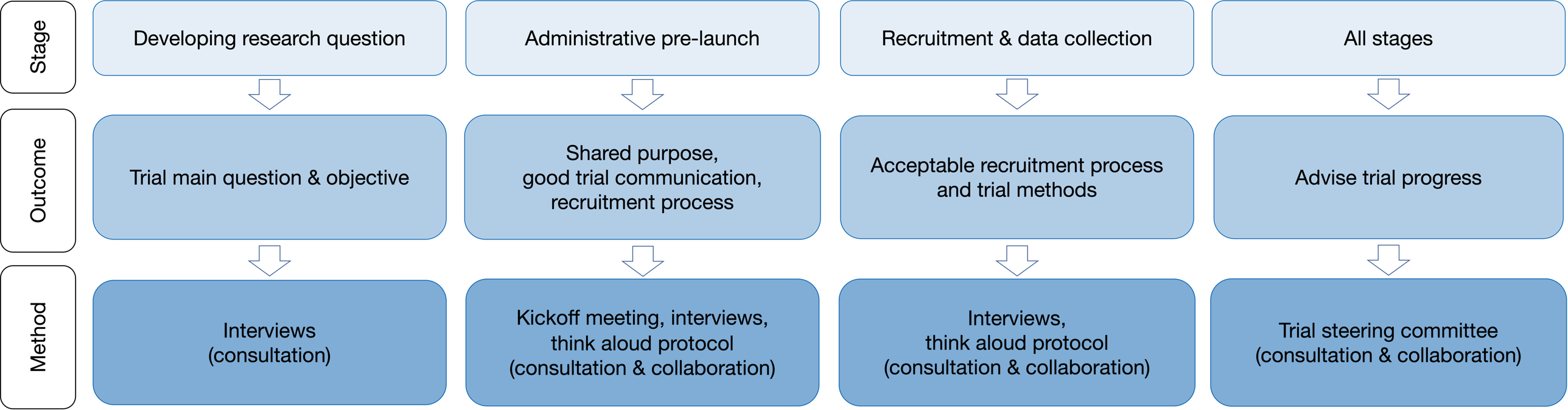
**

Dillon EC, Tuzzio L, Madrid S, Olden H, Greenlee RT. Measuring the impact of patient-engaged research: how a methods workshop identified critical outcomes of research engagement. J Patient Cent Res Rev. 2017 Nov 6;4(4):237–46.
