# Supplementary material for: Involving patients and clinicians in the development of a randomised clinical trial protocol to assess spinal manual therapy versus nerve root injection for patients with lumbar radiculopathy: a patient and public involvement project to inform the SALuBRITY trial design
Source: Res Involv Engagem. 2024 Jan 17;10:8. doi: 10.1186/s40900-023-00536-0 (PMC10792780; doi:10.1186/s40900-023-00536-0)
Supplement: Supplementary file 3 — Additional file 3. Table S2: Themes, categories, and definitions with summarized coding examples. [file 40900_2023_536_MOESM3_ESM.docx]

## Additional file 3: eTable 2. Themes, categories, and definitions with summarized coding examples

Ryf C, Hofstetter L, Clack L, Hincapié CA. Involving patients and clinicians in the development of a randomised clinical trial protocol to assess spinal manual therapy versus nerve root injection for patients with lumbar radiculopathy: a patient and public involvement project to inform the SALuBRITY trial design (2023).

## eTable 2. Themes, categories, and definitions with summarized coding examples

| **Theme** | **Category** | **Sub-category** | **Definition** | **Summarized coding examples** | **Advisor** | **Desired Outcomes** |
| --- | --- | --- | --- | --- | --- | --- |
| Needs | Perception of relevance |  | Added values by conducting study, effect of dissemination of study results | Comprehensive, objective, evidence-informed patient counselling Personalized treatment plan  Short term treatment options  Creating awareness of chiropractic treatment  Alternative access to spinal segmental therapy  No evidence regarding difference of the two treatment options  Weak evidence for spinal manual therapy | P & C  P  C | - Evidence-based patient counselling - Awareness for chiropractic treatment - Evidence for treatment |
| Evidence | Best clinical practice |  | Clinical practice depends on regulations, expert opinion, or experience | Success of nerve root injections during escalation of treatment  SAMM guidelines contraindicate for manipulation during acute radiculopathy Effect of age on treatment response | C | - Revision of current best clinical practice - Guideline development |
| Prerequisites | Request of  pre-study-enrolment information | Procedure related information | Information prior to study enrolment regarding study procedure (blinding, randomization…) | Good education about blinding process and discontinuation of medication  Possibility to exit study  Experienced treatment chiropractor  Randomization is tricky, one should have both options PEG scale explanation of “average” and provide examples for “pain influence on daily activity” | P  C | - Study participation - Lowering occurrence of problems during study participation - Detailed study information in patient information document |
|  |  | Treatment related information | Information prior to study enrolment regarding exposure to treatment | Fast treatment start  Study details with treatment options  Minimizing exposure to unnecessary treatment (CT sham nerve root injections)  Preventing potential negative side effects Information about varying response to nerve root injections | C  P |  |
|  |  | Context related information | Information relevant for recruiting clinician | Contraindication for therapies or medication during follow up while study participation | C | - Patient-centred care during study participation |
|  | Perception of participation terms | Treatment demands | Therapy prerequisites that must be considered for study participation | Patient well-being comes first  Effective treatment  Equal treatment options  No experimental treatment  No disadvantages  Possibility to change treatment method Prevent unnecessary exposure to radiation (CT sham nerve root injections) | P  C | - Patient therapy prerequisites (no experimental treatment, equal options) - Study participation - Adaptations to patient information document |
|  |  | Patient care | Patient care throughout study participation | No restrictions for continued care of recruiting clinician  One contact person for patients during participation  Detailed pain anamnesis  Confidence in caregiver  Intensive care, especially during discontinuation of medication Experienced supervising chiropractor  Importance of patient-cantered care | C  P  C | - Recruiting clinician - Patient-centred care during study participation - Good patient-clinician relationship |
|  | Perception of costs |  | Additional costs added to patient or clinician due to study involvement | Financial impact because of loss of follow up treatments  Cost coverage during treatment?  Added workload for patients | C | - Willingness for study participation or acting as recruiting clinician - Minimization of costs |
| Study procedure | Time management | Request for time efficiency | Temporal requests for increasing efficiency and minimizing patient and clinician burden of participation | Minimal waiting time for patients  Pre-selecting patient with MRI at GP prior to assignment to study  Easy process for recruitment | C | - Adaptations to recruitment process (e.g., pre-selection, minimal effort recruitment) |
|  |  | Prioritization | Prioritization depending on patients’ condition | Screening for categorization of pain intensity  Selection of patients NRS 5-6 as higher level require medication Severe pain requires immediate start of treatment | C  C  P | - Efficient patient recruitment - Adaptations to inclusion criteria (e.g., NRS) |
|  |  | Urgency treatment | Perceived urgency of treatment start | Depending on pain intensity  Assignment as soon as possible  0-5days ambitioned but reasonable  < 5 days for severe pain  > 5 days for chronic patients or for reflection time (cope with diagnosis) | P  C  P | - Range of trial recruitment time (up to 5 days, > 5 days for chronic radiculopathy) - Recruitment time depending on pain intensity |
|  | Perception of outcome recording | Time management | Temporal aspects of recording outcomes along course of treatment | Track pain over course of treatment   - 12 weeks for mobility and psychological effect - 1 year follow up   Varying preference of outcome documentation frequency (daily, weekly, only when in pain) | P | - Outcome assessment along course of treatment - Evaluation of patient preferences for outcome documentation frequency |
|  |  | Documentation | Information about outcome documentation procedure | Pain diary - daily documentation to get into routine  Confirmation of data collection via SMS  Weak medication also bias outcomes  Combination of numeric and visual scale for outcome recording | P  C | - Consideration of pain diary - Documentation of all medication intake during study - Adaptation to outcome recording scale |
|  |  | Clinical relevance | Clinical relevance of outcome, clinical threshold of outcome measure, target of therapy | Pain intensity and its’ influence on daily activity and QoL  Reduced pain radiation  Regain mobility  Psychological stress  Leg and back pain as a measure of QoL Non-inferiority margin of 0.75 points on PEG scale | P  P & C  P  C | - Mobility as outcome measure - Assessment of psychological stress - Confirmation of non-inferiority margin of 0.75 points on PEG scale |
| Patient management | Perception of challenge | Study feasibility | Potential cause of problem requiring feasibility testing | Expectations of treatment effect (e.g., immediate pain relief nerve root injections)  Applicability of treatment (spinal manual therapy possible for severe pain?)  All treatment options should be available  Patient preference impact randomization  Blinding process nerve root injections (feeling of warmth is missing) | P  C | - Balance treatment expectations - Adaptations for inclusion criteria (naive to treatment) - Cross-over study protocol (e.g., applicability of therapy) |
|  |  | Patient compliance | Patient compliance regarding medication intake and use of other therapies | Documentation of medication intake  Control for medication and other treatment options | C | - Documentation of patient activities outside study |
|  |  | Call of emergency | Any kind of emergency during study enrolment requiring immediate action | Treatment escalation and cross-over study protocol  Possibility to exit study  Emergency medication | P | - Treatment escalation protocol - Emergency medication protocol |
|  | Pain management | Perception of pain | Location of perceived pain and characteristics regarding measure of treatment effect | Back pain  Leg pain for radiculopathy  Overall pain to cover all people  Ask leg and back pain separately – very individual  Average but also peak pain relevant | P  P & C  P & C  C  P | - Individual location of perceived pain - Individual outcome measures - Average & peak pain |
|  |  | Perception of the ability to suffer | Willingness for discontinuation of pain medication | Depending on patient tolerance,  pain intensity (specially for 48h), patient context (e.g., responsibilities family, work), acceptance of short term restriction  Consider no discontinuation due to  pragmaticism and relevance of long term outcome  Discontinuation for 12-24h acceptable | C  P  C  P & C | - Range of medication discontinuation: 12-24h |
|  |  | Pain relief | Techniques for pain relief, patients’ attitude towards pain medication | Willingness for alternative pain treatment (e.g., cold)  Main thing is pain relief for severe pain  Level schema emergency medication | C  P | - Alternative pain treatment during medication discontinuation - Documentation of medication and alternative therapies |
| Communication | Feedback Request |  | Feedback regarding referred patient and study updates | Study update regarding patient condition as soon as possible  Study results Study results for evidence-based patient counselling | C  P | - Regular feedback about patient condition to recruiting clinician (e.g., GP) - Dissemination of study results |
|  | Establishment of recruitment network | Efficient recruitment | Prerequisite for efficient recruitments | Monthly study reminder for recruitment  Patient recruitment via confidant (GP)  Personal contact to recruiting clinician before communication via mail  Clinician information document and study abstract for recruiting clinician  Simple recruitment process | C  P  C | - Study reminder - Establish personal contact with recruiting clinician |
|  |  | Location | Potential places for patient recruitment, collaboration contacts | Emergency centre  Rheumatology USZ  Institute for GP UZH  Joint practices surrounding area  SAMM GPs (could also be critical due to guidelines)  Quality circles  MediDays Zurich | C | - Collaboration contacts - SAMM GP require special attention in recruitment process |
| *Abbreviations: SAMM = Schweizerische Ärztegesellschaft für Manuelle Medizin, CT = computer tomography, MRI = magnetic resonance imaging, GP = general practitioner, NRS = numeric rating scale, QoL = quality of live, PEG scale = pain, enjoyment of life and general activity scale.* | | | | | | |
