# Supplementary material for: Involving patients and clinicians in the development of a randomised clinical trial protocol to assess spinal manual therapy versus nerve root injection for patients with lumbar radiculopathy: a patient and public involvement project to inform the SALuBRITY trial design
Source: Res Involv Engagem. 2024 Jan 17;10:8. doi: 10.1186/s40900-023-00536-0 (PMC10792780; doi:10.1186/s40900-023-00536-0)
Supplement: Supplementary file 4 — Additional file 4. Figure S2: Shared purpose statement. [file 40900_2023_536_MOESM4_ESM.docx]

## Additional file 4: eFigure 2. Shared purpose statement (German and English translation)

Ryf C, Hofstetter L, Clack L, Hincapié CA. Involving patients and clinicians in the development of a randomised clinical trial protocol to assess spinal manual therapy versus nerve root injection for patients with lumbar radiculopathy: a patient and public involvement project to inform the SALuBRITY trial design (2023).

## eFigure 2. Shared purpose statement (Original German)

**Shared purpose statement** (English translation)

Dear project partner

Thank you for your participation in today's kick-off event. It was wonderful to see all project partners united on "one screen." We have provided you with a lot of information about the study and hope that you are now well-prepared and confident enough to start the individual or group interviews. We look forward to your inputs and trust that you are still motivated to share your experience and perspective as a patient or clinician on selected questions of the SALuBRITY study.

To define a common PPI project goal, a so-called "shared purpose," we asked about your expectations and goals during our meeting, leading to the following results:

- Gain insight into research
- Exchange personal life experiences of patients with lumbar radiculopathy and treating clinicians
- Patient-oriented perspectives on treatment options
- Openness to alternative treatment methods
- Comparison of the indication and effectiveness of treatment methods
- Dissemination of new findings

If you do not agree with the above-mentioned goals or have any additions, please feel free to contact leonie.hofstetter@balgrist.ch.

We look forward to further discussions and hope that, with your help, we can improve the quality and relevance of the planned SALuBRITY study.
